# Mitochondrial DNA Depletion in Granulosa Cell Derived Nuclear Transfer Tissues

**DOI:** 10.3389/fcell.2021.664099

**Published:** 2021-05-14

**Authors:** Daniela Bebbere, Susanne E. Ulbrich, Katrin Giller, Valeri Zakhartchenko, Horst-Dieter Reichenbach, Myriam Reichenbach, Paul J. Verma, Eckhard Wolf, Sergio Ledda, Stefan Hiendleder

**Affiliations:** ^1^Department of Veterinary Medicine, University of Sassari, Sassari, Italy; ^2^Molecular Animal Breeding and Biotechnology, Gene Center and Department of Veterinary Science, LMU Munich, Munich, Germany; ^3^ETH Zürich, Animal Physiology, Institute of Agricultural Sciences, Zurich, Switzerland; ^4^Bavarian State Research Center for Agriculture, Institute of Animal Breeding, Grub, Germany; ^5^Bayern-Genetik GmbH, Grub, Germany; ^6^Livestock Sciences, South Australian Research and Development Institute, Roseworthy, SA, Australia; ^7^School of Animal and Veterinary Sciences, The University of Adelaide, Roseworthy, SA, Australia; ^8^Davies Research Centre, School of Animal and Veterinary Sciences, The University of Adelaide, Roseworthy, SA, Australia; ^9^Robinson Research Institute, The University of Adelaide, Adelaide, SA, Australia

**Keywords:** nuclear transfer, mitochondrial DNA depletion, mitochondrial gene expression, fetal tissues, bovine

## Abstract

Somatic cell nuclear transfer (SCNT) is a key technology with broad applications that range from production of cloned farm animals to derivation of patient-matched stem cells or production of humanized animal organs for xenotransplantation. However, effects of aberrant epigenetic reprogramming on gene expression compromise cell and organ phenotype, resulting in low success rate of SCNT. Standard SCNT procedures include enucleation of recipient oocytes before the nuclear donor cell is introduced. Enucleation removes not only the spindle apparatus and chromosomes of the oocyte but also the perinuclear, mitochondria rich, ooplasm. Here, we use a Bos taurus SCNT model with in vitro fertilized (IVF) and in vivo conceived controls to demonstrate a ∼50% reduction in mitochondrial DNA (mtDNA) in the liver and skeletal muscle, but not the brain, of SCNT fetuses at day 80 of gestation. In the muscle, we also observed significantly reduced transcript abundances of mtDNA-encoded subunits of the respiratory chain. Importantly, mtDNA content and mtDNA transcript abundances correlate with hepatomegaly and muscle hypertrophy of SCNT fetuses. Expression of selected nuclear-encoded genes pivotal for mtDNA replication was similar to controls, arguing against an indirect epigenetic nuclear reprogramming effect on mtDNA amount. We conclude that mtDNA depletion is a major signature of perturbations after SCNT. We further propose that mitochondrial perturbation in interaction with incomplete nuclear reprogramming drives abnormal epigenetic features and correlated phenotypes, a concept supported by previously reported effects of mtDNA depletion on the epigenome and the pleiotropic phenotypic effects of mtDNA depletion in humans. This provides a novel perspective on the reprogramming process and opens new avenues to improve SCNT protocols for healthy embryo and tissue development.

## Introduction

Somatic cell nuclear transfer (SCNT) is a platform technology with a broad spectrum of current and future applications. These include multiplication of agriculturally important genetics to improve livestock, generation of animal models for human diseases, production of pharmaceuticals and xenotransplants, and reprogramming of human somatic cells into pluripotent embryonic stem cells (SCNT-ESCs) for patient-matched cell therapies (Niemann and Lucas-Hahn, 2012; Matoba and Zhang, 2018). The successful production of SCNT-ESCs from human embryos (Tachibana et al., 2013) and the cloning of monkeys (Liu et al., 2018), combined with significant advances in gene editing technology (Tan et al., 2016), have further boosted interest in SCNT technology and its current and future applications.

More than three decades after inception (Campbell et al., 1996; Wilmut et al., 1997), SCNT is still fraught with low success rates and a high frequency of phenotypic abnormalities, which have been ascribed to aberrant epigenetic reprogramming of the somatic donor nucleus and impaired epigenetic status of SCNT embryos (Kang et al., 2001; Santos et al., 2003; Beaujean et al., 2004; Chan et al., 2012; Zhang et al., 2016), fetuses (Hiendleder et al., 2004a; Liu et al., 2008; Zhang et al., 2014), and offspring (Xue et al., 2002; de Montera et al., 2010; Shen et al., 2013). However, considering that SCNT is typically based on oocyte enucleation followed by insertion of a nuclear donor cell with foreign mitochondria (Campbell et al., 1996), and the central roles of mitochondria in energy production, cell signaling, and growth and development, mitochondrial perturbations have long been suspected to contribute to SCNT failures (Hiendleder et al., 2003, 2005).

Varying degrees of donor cell introduced heteroplasmy that disrupt the uniparental mode of mitochondrial DNA (mtDNA) inheritance in mammals (Hiendleder, 2007) and the specific effects of heterologous mtDNA haplotypes in cytoplast–donor cell combinations (Bruggerhoff et al., 2002; Hiendleder et al., 2004b; Jiao et al., 2007; Yan et al., 2010) were the focus of earlier studies to elucidate mitochondrial effects on SCNT outcomes. Nonetheless, the degree of mtDNA heteroplasmy after SCNT is generally low (Hiendleder, 2007), and the majority of SCNT experiments failed to detect advantages in embryo development when homoplasmy was preserved (Edwards et al., 2003; Murakami et al., 2003; Yang et al., 2006; Lee and Song, 2007). Furthermore, in the non-SCNT mouse model, high levels of artificial heteroplasmy almost exclusively affect behavior phenotypes (Sharpley et al., 2012), with no evidence for increased abnormality and mortality rates (Jenuth et al., 1996; Battersby et al., 2003; Sharpley et al., 2012) commonly associated with SCNT (e.g., Rhind et al., 2003; Sakai et al., 2005; Hill, 2014). Similarly, phenotypic effects of different mtDNA–nuclear DNA (nDNA) combinations in backcrossed mice appear to be limited to brain morphology and behavior phenotypes (Roubertoux et al., 2003). It is therefore unlikely that mtDNA heteroplasmy or mtDNA–nDNA incompatibility accounts for a significant proportion of the losses and aberrant phenotypes observed in intraspecies SCNT.

Perturbation of mitochondria and mtDNA content through removal of mitochondria-rich perinuclear cytoplasm (Nagai et al., 2006) and disruption of the oocyte cytoskeleton by SCNT procedures (Katayama et al., 2006) provide an alternative pathway for mitochondrial effects on the development of reconstructed embryos. The fundamental importance of mtDNA for pre- and postnatal development is clearly demonstrated by mtDNA depletion syndromes in humans that are characterized by a tissue-specific reduction in mtDNA and respiratory deficiencies that manifest in a range of phenotypes, including hydrops, tendon and limb deformities, hepatomegaly, hypertrophic cardiomyopathy, nephropathy, muscle hypotonia, respiratory distress, and lactic acidosis (von Kleist-Retzow et al., 2003; Sarzi et al., 2007; Suomalainen and Isohanni, 2010; El-Hattab and Scaglia, 2013; Finsterer and Ahting, 2013; Nogueira et al., 2014). These mtDNA depletion phenotypes show striking similarities with and may be identical to developmental abnormalities observed after SCNT (e.g., Rhind et al., 2003; Chavatte-Palmer et al., 2004; Wells et al., 2004; Sakai et al., 2005; Brisville et al., 2013; Hill, 2014). Importantly, mouse models with mtDNA depletion have clearly demonstrated significant detrimental effects of reduced mtDNA amount on embryo survival and organogenesis (Larsson et al., 1998; Huo and Scarpulla, 2001; Hance et al., 2005). Nevertheless, mtDNA quantity in postimplantation SCNT tissues has, to our knowledge, not been studied. Here, we use a day 80 (29% term) Bos taurus model to demonstrate the deleterious effects of SCNT procedures on mtDNA amount and mitochondrial gene expression that are associated with hallmarks of an SCNT-specific overgrowth phenotype.

## Materials and Methods

### Ethics Statement

Fetuses were not specifically generated for this study, as all samples were obtained from a tissue bank for day 80 B. taurus fetuses that were used in previous studies (e.g., Hiendleder et al., 2003, 2004a,2004b). All experiments involving animals were performed in accordance with the relevant guidelines for the care and use of animals and with approval by the responsible animal welfare authority, the Regierung von Oberbayern (Bavaria, Germany).

### Fetuses

We used samples from AI (n = 24), IVF (n = 21), and SCNT (n = 22) fetuses that consisted of males and females, singletons and nonsingletons, with Brown Swiss and Simmental genetics. This data set is referred to as the entire data set. In order to confirm key findings, we analyzed two stringently defined core subsets that consisted of samples from singleton Brown Swiss fetuses only. The AI and IVF fetuses in the core subsets had the same sire, a son of the nuclear donor cow for SCNT fetuses. We were thus able to perform highly standardized comparisons between female SCNT (n = 14) and AI (n = 9) and between male and female IVF (n = 9) and AI (n = 13), tissues. A detailed description of samples is presented in Supplementary Table 1.

### Oocyte Maturation for SCNT and IVF

Unless otherwise indicated, chemicals and reagents used in experiments were purchased from Sigma Chemical Co. (St. Louis, MO, United States). Briefly, cumulus–oocyte complexes (COCs) were obtained by aspiration from Brown Swiss and Simmental ovaries, washed in TCM 199 Hepes (Seromed, Berlin, Germany), and transferred to four-well plates (Nunc, Roskilde, Denmark) with 400 μl modified Parkers medium (MPM) containing TCM199 (Biochrom, Berlin, Germany) supplemented with L-glutamine (100 mg/L), NaHCO_3_ (800 mg/L), HEPES (1400 mg/L), sodium-pyruvate (250 mg/L), L-lactic-calcium-salt (600 mg/L), gentamicin (55 mg/L, Seromed), and 10% estrous cow serum (ECS) containing 0.01 U/ml b-FSH and b-LH (Sioux Biochem, Sioux Center, IA, United States) for SCNT and 0.2 U/ml o-FSH (Ovagen, ICPbio, Auckland, New Zealand) for IVF. Brown Swiss and Simmental oocytes for SCNT were matured for 18 h at 39°C in an atmosphere of 5% CO_2_ with maximum humidity, incubated for 5 min in modified phosphate-buffered saline [mPBS; PBS plus 4 mg/ml bovine serum albumin (BSA)] containing 3 mg/ml hyaluronidase, vortexed for 4 min, and stripped of cumulus cells by gentle pipetting. Oocytes for IVF were matured for 20–22 h at 39°C in an atmosphere of 5% CO_2_ and maximum humidity.

### Nuclear Donor Cell Preparation

Granulosa cells collected from a Brown Swiss cow were washed twice in saline solution, dispersed in 0.1% (w/v) trypsin (Gibco, Grand Island, NY, United States), and transferred to 5-cm culture dishes with Dulbecco’s modified Eagle’s medium (DMEM) (Gibco) supplemented with 10% (v/v) fetal calf serum (FCS) (Biochrom, Berlin, Germany), 2 mM L-glutamine, 0.1 mM β-mercaptoethanol, 2 mM nonessential amino acids, 100 IU/ml penicillin, and 100 μg/ml streptomycin. Cells were cultured until subconfluence at 37°C in a humidified atmosphere of 5% CO_2_ in air, frozen in 10% (v/v) dimethylsulfoxide in FCS, and stored in liquid nitrogen. For SCNT experiments, cells were thawed and cultured for three to six passages until confluence just before SCNT.

### Somatic Cell Nuclear Transfer and Culture of Embryos

Somatic cell nuclear transfer was performed at 20–25°C with Leitz micromanipulators (Leica Microsystems, Wetzlar, Germany) and a Wilovert stereo microscope. Oocytes with polar body were placed in mPBS containing 5 μg/ml cytochalasin B and incubated for 5–10 min before enucleation. Enucleation was performed in a small drop of mPBS in a micromanipulation chamber by aspirating the polar body with a small volume of surrounding cytoplasm into a micropipette. Oocytes were stained with 2 μg/ml Hoechst 33342 dye and assessed by an epifluorescence microscope (Zeiss, Jena, Germany) to confirm enucleation. A donor cell was then transferred into the perivitelline space and the resulting karyoplast–cytoplast complex (KCCs) exposed to a double electric pulse of 2.1 kV/cm for 10 μs using the Zimmermann Cell Fusion Instrument (Bachofer, Reutlingen, Germany). The KCCs were placed in the incubator in Ham F-12 medium supplemented with 0.3% BSA and activated by a 5-min incubation in 7% ethanol 2 h postfusion followed by 5 h culture in 10 μg/ml cycloheximide and 5 μg/ml cytochalasin B (E-Chx). This was followed by three washes in culture medium and transfer into 100 μl drops of synthetic oviduct fluid medium (SOF) supplemented with 2% basal medium Eagle (BME) amino acids (Gibco), 1% minimum essential medium (MEM) nonessential amino acids (Gibco), and 10% (v/v) ECS. The SOF drops were covered with paraffin oil (Merck, Darmstadt, Germany) and reconstructed embryos cultured at 39°C in a humidified atmosphere of 5% CO_2_, 5% O_2_, and 90% N_2_. Fetuses were collected from a total of 14 SCNT sessions.

### In vitro Fertilization and Culture of Embryos

Matured COCs were washed three times in fertilization medium (Tyrode albumin lactate pyruvate) supplemented with sodium pyruvate (2.2 mg/ml), heparin sodium salt (2 mg/ml), and BSA (6 mg/ml) and transferred to 400 μl droplets of medium. Frozen–thawed spermatozoa that had been subjected to the swim-up procedure for 90 min were coincubated with COCs at 2 × 10^6^ cells/ml for 18 h in maximum humidity, 39°C, and 5% CO_2_ in air. Presumptive zygotes were mechanically denuded by vortexing, washed three times in SOF culture medium with 2% BME (Invitrogen, Karlsruhe, Germany), 1% MEM (Invitrogen), and 10% ECS and transferred to 400 μl droplets of medium covered with mineral oil. The culture atmosphere was 5% CO_2_, 5% O_2_, 90% N_2_, and 39°C at maximum humidity.

### Embryo Transfer and Artificial Insemination

On day 7 after SCNT and IVF, all viable embryos were transferred nonsurgically to synchronous Simmental recipient heifers. Insemination of heifers with frozen–thawed semen was performed by standard procedures. In order to match the transfer of more than one embryo in SCNT and IVF experiments, a subgroup of heifers for collection of AI fetuses was mildly stimulated hormonally before AI to induce twinning. Heifers received 500 IE ECG (Intergonan, Intervet, i.m.) between days 9 and 13 of the estrous cycle followed by 2 ml PGF2α (Estrumate, Intervet, i.m.) 60 h later. AI was performed after 48–60 h. Pregnancies were confirmed on day 28 by ultrasonographic examination and on day 79 by palpation.

### Samples and Phenotype Data

Fetuses were recovered at day 80 post conception after recipient heifers were humanely killed in an abattoir. Fetal weight and dimensions, including crown-rump length (CRL) and thorax circumference (TC), and absolute and relative liver weight were recorded. All fetuses were from viable intact pregnancies.

To capture potential differences in mtDNA content or gene expression that originate early in development, we used tissues representing the three germ layers ectoderm (brain), endoderm (liver), and mesoderm (skeletal muscle).

Brain samples were obtained from the upper-left cerebral hemisphere, liver samples from the Lobus hepatis sinister, and skeletal muscle samples from the left Musculus biceps femoris region. All tissue samples were collected on ice. Samples for DNA extraction were snap frozen in liquid nitrogen and those for RNA extraction placed in RNAlater (Ambion, Austin, United States) and stored at −80°C after 24 h at 4°C. For assessment of muscle mass, the spinal column with cervical, thoracic, and lumbar vertebrae and the left and right sixth rib were removed from the fetal carcass, cleaned of tissue (Xiang et al., 2014), and their lengths measured using photographic images and the image analysis program analySIS (analySIS, IBM, St Leonards NSW, Australia).

### Quantitation of Mitochondrial DNA

Total DNA was extracted from fetal brain, liver, and muscle tissue with the E.Z.N.A. Tissue DNA Mini Kit II including RNase A treatment (PEQLAB Biotechnologie GmbH, Erlangen, Germany) and quantified by repeated spectrophotometry of samples.

A conserved 176 nt segment (GenBank no. V00654) of the mtDNA control region was amplified from 5 ng DNA with primers 5′-ACACAGAATTTGCACCCTAACC-3′ and 5′-GCCCCATGCATATAAGCAAG-3′ using a Light-Cycler and the LightCycler-FastStart DNA Master SYBR Green I Kit (Roche Diagnostics, Mannheim, Germany) with the following conditions: annealing temperature (AT), 60°C; melting point (MP), 80°C; and fluorescence acquisition (FA), 77°C. Quantification cycles (Cq) were calculated with the second derivative maximum method (LightCycler software version 3.5.28). The mtDNA Cq was normalized against a 365-nt segment (GenBank no. NR_036642) of nuclear RNA18S gene (5′-AAGTCTTTGGGTTCCGGG-3′ and 5′-GGACATCTAAGGGCATCACA-3′; AT, 60°C; MP, 90°C; FA, 87°C). Amplified fragments were verified by gel electrophoresis and sequencing. Primers were from Microsynth, Balgach, Switzerland.

### Gene Expression Analyses

Total RNA for gene expression analyses was extracted from 100 to 200 mg of tissue as described (Chomczynski and Sacchi, 1987) using Trizol (Invitrogen, Karlsruhe, Germany). After DNase I treatment (Invitrogen, Karlsruhe, Germany), RNA concentration was measured by a spectrophotometer and integrity determined by gel electrophoresis. One microgram of each RNA sample was reverse transcribed in a total volume of 60 μl: 5× buffer (Promega), 10 mM deoxyribonucleotide triphosphates (dNTPs) (Roche, Mannheim, Germany), 50 μM hexamers (Gibco-BRL, Grand Island, United States), and 200 U Superscript RT enzyme (Promega, Madison, United States).

Real-time quantitative PCR (qPCR) to quantify expression levels of reference genes UBB, H3F3A, and YWHAZ and target genes MT-ND1, MT-CYTB, MT-COX3, MT-ATP8, POLGA, POLGB, and TFAM was performed with the KAPA SYBR FAST qPCR Kit (Kapa Biosystems, Wilmington, United States) on a CFX384 Real-Time PCR Detection System (Bio-Rad, Munich, Germany). Cq-values were obtained using a single threshold. Details of all primers (Microsynth, Balgach, Switzerland) and amplicons are presented in Supplementary Table 2. Expression of target genes was normalized against the geometrical mean of expression of three reference genes. Fragments were verified by gel electrophoresis and sequencing.

All qPCR reactions were performed in duplicate. For each primer pair, the efficiency of the PCR was determined by building a standard curve with serial dilutions of a known amount of template, covering at least three orders of magnitude so that the calibration curve’s linear interval included the interval above and below the abundance of the targets. All primers achieved an efficiency of reaction between 90 and 110% (3.6 > slope > 3.1) and a coefficient of determination (R^2^) > 0.99.

### Statistical Analyses

We used the general linear model procedure of IBM SPSS Statistics version 24 (IBM Corp., Armonk, NY, United States) and performed ANOVA to determine effects of treatment on investigated parameters.

We analyzed the entire fetal dataset with the model

Yijk=Ti+Sj+Pk+eijk

where Yijk is the measured parameter, T(*i* = SCNT, AI, IVF) is the treatment, S(*j* = female, male) is the sex effect, P(*k* = singleton, nonsingleton) is the pregnancy type, and eijk is the random error.

To determine differences between SCNT and AI groups in the more stringently defined fetal core subset consisting of female singleton fetuses with Brown Swiss genetics only, we used the model

Yi=Ti+ei

where Yi is the measured parameter, T(*i* = SCNT, AI) is the treatment effect, and ei is the random error.

To test for potential effects of *in vitro* embryo culture in a stringently defined fetal core subset consisting of female and male singleton fetuses with Brown Swiss genetics, we used the model

Yij=Ti+Sj+eij

where yij is the measured parameter, T(*i* = IVF, AI) is the treatment, S(*j* = female, male) is the sex effect, and eij is the random error.

Least squares means with standard errors of means for fetal parameters were computed and compared using two-tailed *t*-test with a significance threshold of *P* < 0.05.

Regressions and correlation coefficients were calculated with GraphPad Prism 7.02 (GraphPad Software, La Jolla, CA, United States) with a significance threshold of *P* < 0.05 and graphs produced with the same software.

## Results

### Abnormal SCNT Phenotype

**FIGURE 1:**
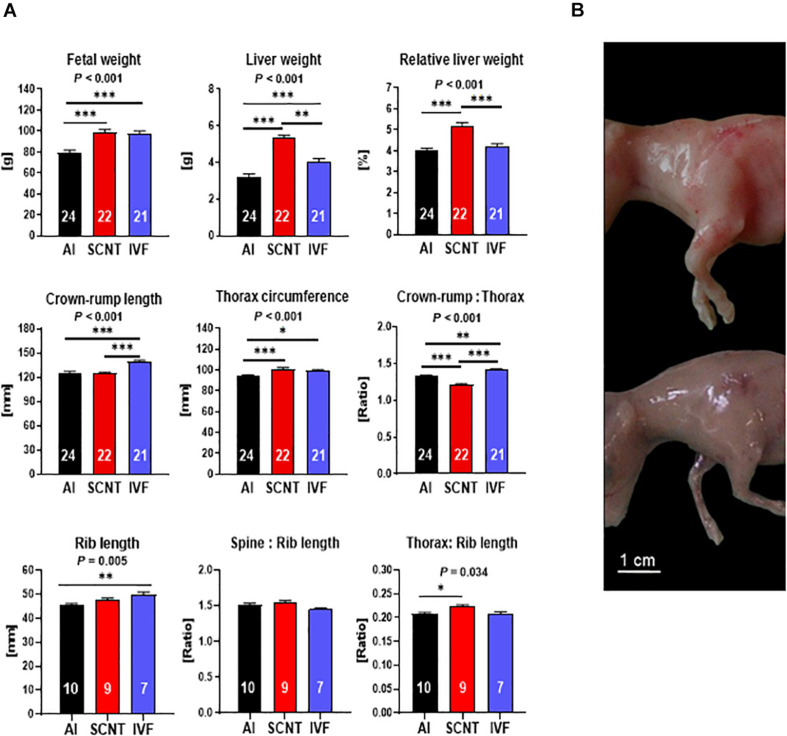
Abnormal phenotype after somatic cell nuclear transfer (SCNT) involves hepatomegaly and muscle hypertrophy. **(A)** Effects of SCNT on fetal phenotype as compared with *in vitro* fertilization (IVF) and artificial insemination (AI) controls at day 80 of gestation. Crown rump:thorax is crown-rump length to thorax circumference ratio, rib length is the combined length of both sixth ribs, spine:rib length is the ratio of the length of the spinal column to the combined length of both sixth ribs, and thorax:rib length is the ratio of thorax circumference to combined length of both sixth ribs. Means ± standard error of the mean (SEM) and *P*-values for significant effects of treatment (AI, IVF, and SCNT) on phenotypic parameters in ANOVA (*F*-test) are shown. Significant differences between group means are indicated by asterisks (*t*-test; ^∗^*P* < 0.05, ^∗∗^*P* < 0.01, ^∗∗∗^*P* < 0.001). The number of fetuses analyzed for each parameter is given inside bars. **(B)** SCNT fetus displaying typical phenotype with increased muscle mass (top) compared to AI control (bottom).

SCNT fetuses were heavier (+25.9%, *P* < 0.001) and had higher absolute (+66.0%, *P* < 0.001) and relative (+32.1%, *P* < 0.001) liver weights than AI controls (Figure 1A). Morphometric analyses revealed that CRL of SCNT fetuses was similar to AI controls (*P* > 0.05), but thorax circumference (TC) was increased (+7.9%, *P* < 0.001) and CRL:TC ratio, therefore, decreased (−8.6%, *P* < 0.001). These data could be explained by (i) a disproportionate general increase in TC or (ii) increased muscle mass in the TC region. Comparative visual assessment of SCNT and AI fetuses provided strong support for the latter explanation (Figure 1B). However, the lack of definition of individual muscles at the day 80 fetal stage precluded a direct analysis of muscle mass. We therefore opted for an indirect assessment of muscle mass and removed all muscle tissue from the spinal column and the left and right sixth rib for comparison of skeletal dimensions with external measurements of fetuses. The combined length of both ribs, analogous to TC measurement, and the ratio of spinal column length to combined rib length, analogous to CRL:TC ratio, were indeed similar (both *P* > 0.05) for SCNT and AI fetuses. Furthermore, TC to rib length ratio in SCNT fetuses was higher than that in AI fetuses (+7.4%, *P* = 0.001), indicating a higher muscle mass (Figures 1A,B). Increased TC and decreased CRL:TC ratio of SCNT fetuses is therefore caused by increased muscle mass and not by a general increase in TC.

Although IVF control fetuses were heavier (+20.1%, *P* = 0.006) and had higher absolute (+23.7%, *P* < 0.001) liver weight than AI controls (Figure 1A), their relative liver weight was similar to controls (*P* > 0.05). Furthermore, morphometric analyses revealed an increase in CRL (+11.5%, *P* < 0.001) and TC (+4.7%, *P* < 0.05) of IVF fetuses that resulted in an increased CRL:TC ratio (+6.6%, *P* < 0.05) as compared with AI controls. Skeletal parameters including increased combined rib length (+10.1%, *P* < 0.01) and TC to rib length and spine to rib length ratios similar to AI controls (both *P* > 0.05) further supported a proportionate increase in size but otherwise normal phenotype of IVF fetuses (Figure 1A).

Two core subsets of the entire data set, where only singletons were retained and genetics was more strictly controlled, were used to confirm key findings obtained with the entire data set (see section “Materials and Methods”). Analyses of the SCNT-AI and IVF-AI subsets confirmed hepatomegaly and muscle hypertrophy of SCNT fetuses (Supplementary Figure 1A) and the increased size but otherwise normal phenotype of IVF fetuses (Supplementary Figure 1B).

### Mitochondrial DNA Depletion in SCNT Tissues

**FIGURE 2:**
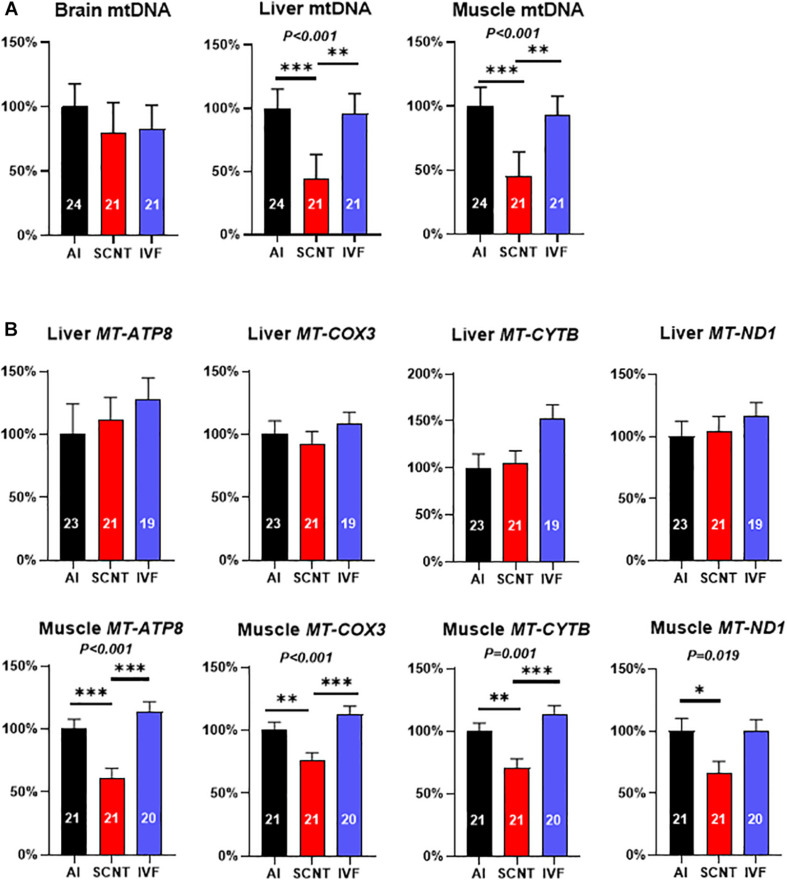
Somatic cell nuclear transfer (SCNT) affects mitochondrial DNA (mtDNA) amount and mitochondrial gene expression. **(A)** Effects of SCNT on relative mtDNA amount of fetal tissues as compared with *in vitro* fertilization (IVF) and artificial insemination (AI) controls at day 80 of gestation. **(B)** Effects of SCNT on transcript abundance of mtDNA genes encoding subunits for respiratory chain enzyme complexes I, III, IV, and V (*MT-ATP8*, *MT-COX3*, *MT-CYTB*, and *MT-ND1*) in liver (top row) and skeletal muscle (bottom row). Means ± standard error of the mean (SEM) and *P*-values for significant effects of treatment (AI, IVF, and SCNT) on phenotypic parameters in ANOVA (*F*-test) are shown. Significant differences between group means are indicated by asterisks (*t*-test; ^∗^*P* < 0.05, ^∗∗^*P* < 0.01, ^∗∗∗^*P* < 0.001). The number of fetuses analyzed for each parameter is given inside bars.

Relative mtDNA amount in the SCNT liver and skeletal muscle was significantly lower than in AI controls (−56.2%, *P* < 0.001 and −54.6%, *P* = 0.001), while mtDNA amount in the SCNT brain was unaffected (*P* > 0.05; Figure 2A). In contrast, comparisons between IVF and AI fetuses indicated similar mtDNA amounts in all three tissues (*P* > 0.05; Figure 2A).

Analyses of the core subsets of fetuses confirmed the significant reduction in mtDNA in the SCNT skeletal muscle (−60.7%, *P* = 0.001; Supplementary Figure 2A), but reduced mtDNA amount in the SCNT liver was no longer significant (−28.20%, *P* > 0.05). As in the entire dataset, mtDNA amounts in all three tissues of IVF fetuses were similar (*P* > 0.05) to AI controls (Supplementary Figure 2B).

### Reduced Mitochondrial Gene Expression in mtDNA-Depleted SCNT Muscle

The mtDNA depletion in the SCNT liver and skeletal muscle could be expected to impact transcript abundance of mtDNA-encoded subunits of respiratory chain enzyme complexes. Quantitation of *MT-ND1* (complex I), *MT-CYTB* (complex III), *MT-COX3* (complex IV), and *MT-ATP8* (complex V) transcripts revealed a significant reduction in all measured transcripts in the SCNT muscle as compared with AI controls (*MT-ATP8*: −38.7%, *P* < 0.001; *MT-COX3*: −23.7%, *P* < 0.01; *MT-CYTB*: −29.4%, *P* < 0.001; *MT-ND1*: −33.4%, *P* < 0.05; Figure 2B), while none of these transcripts were affected in the liver (*P* > 0.05, Figure 2B). All transcript abundances for mtDNA-encoded genes in tissues from IVF fetuses were similar to AI controls (*P* > 0.05, Figure 2B).

Comparisons in the core subsets of fetuses confirmed a significant reduction in *MT-ATP8* (−34.93%, *P* < 0.05) and *MT-ND1* (−35.11%; *P* < 0.05) transcript of the SCNT skeletal muscle, while none of the four transcripts in that tissue were affected by IVF (Supplementary Figures 2C,D). Furthermore, and again in accordance with the entire dataset, none of the four transcripts in the liver was affected by SCNT or IVF (*P* > 0.05; Supplementary Figures S2C,D).

### Expression of Nuclear Genes Involved in mtDNA Replication Is Not Affected by SCNT

**FIGURE 3:**
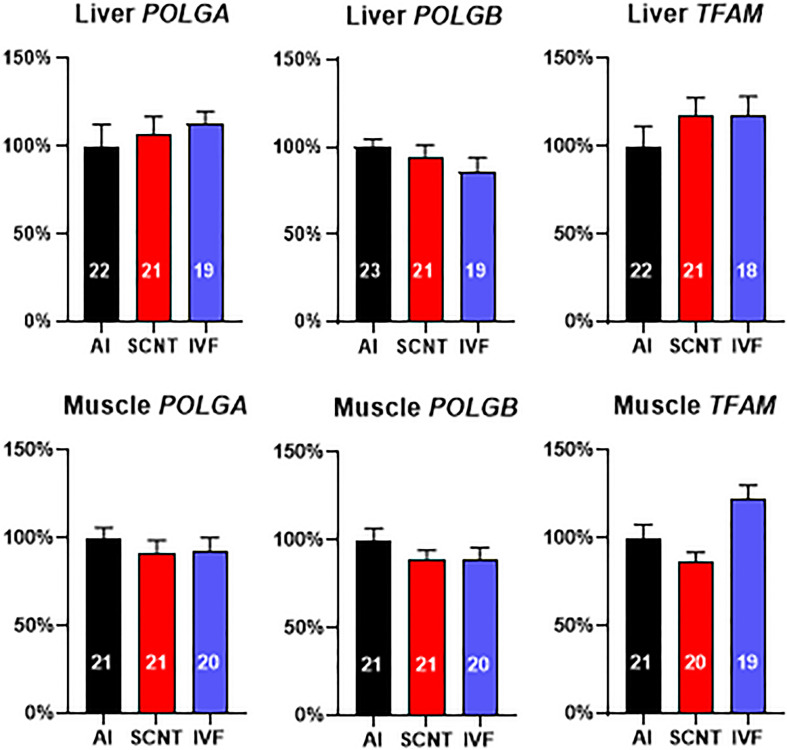
Somatic cell nuclear transfer (SCNT) does not affect the expression of three nuclear-encoded genes pivotal for mitochondrial DNA (mtDNA) replication. Effect of SCNT on *POLGA*, *POLGB*, and *TFAM* transcript abundance in liver and skeletal muscle as compared with *in vitro* fertilization (IVF) and artificial insemination (AI) controls at day 80 of gestation. Means ± SEM are shown; treatment group was not significant (ANOVA general linear model; *P* > 0.05). The number of fetuses analyzed for each parameter is given within bars.

We next examined expression of three nuclear-encoded genes that are pivotal for mtDNA replication and could potentially have been adversely affected by aberrant epigenetic reprogramming. However, SCNT, AI, and IVF fetuses demonstrated similar transcript levels (*P* > 0.05) in the liver and skeletal muscle for subunits of mtDNA polymerase gamma (*POLGA* and *POLGB*) and mitochondrial transcription factor A (*TFAM*) (Figure 3). Analyses of the core subsets of fetuses confirmed results obtained with the entire dataset (Supplementary Figures S3A,B).

### Relationships Between mtDNA Amount and Phenotype

**FIGURE 4:**
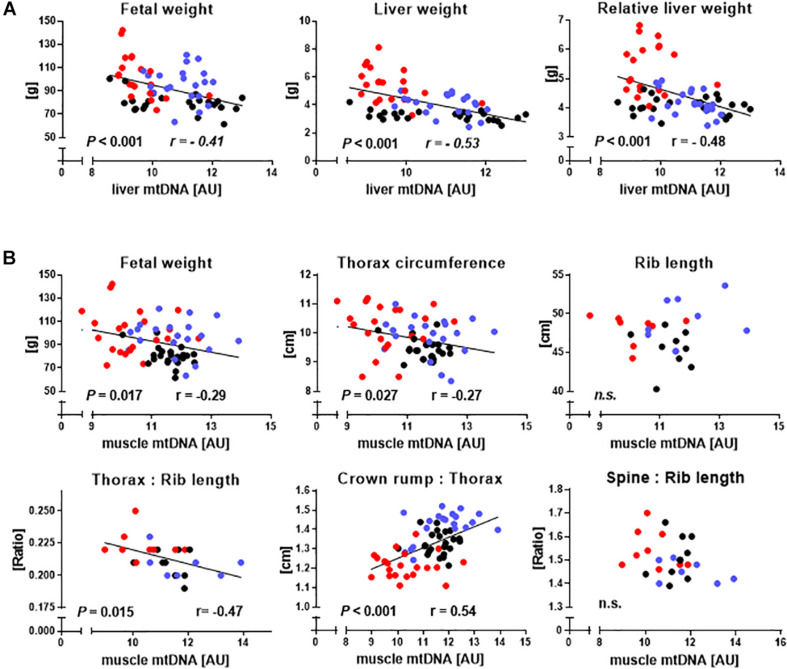
Relationships between hallmarks of somatic cell nuclear transfer (SCNT) specific disproportionate fetal overgrowth and mtDNA amount in liver and skeletal muscle. **(A)** Regressions of fetal weight and absolute and relative liver weight on liver mtDNA amount of somatic cell nuclear transfer (SCNT, red), *in vitro* fertilization (IVF, blue), and artificial insemination (AI, black) fetuses. **(B)** Regression of phenotypic parameters describing increased fetal muscle mass on muscle mtDNA amount. Rib length is the combined length of both sixth ribs, thorax:rib length is the ratio of thorax circumference to combined length of both sixth ribs, crown rump:thorax is crown-rump length to thorax circumference ratio, and spine:rib length is the ratio of the length of the spinal column to the combined length of both sixth ribs. Significant *P*-values and Pearson correlation coefficients (r) are shown. MtDNA amount is expressed in arbitrary units (AU).

We then used the entire dataset to explore relationships between relative mtDNA amount and phenotype (Figures 4A,B). Indicators of liver mass, including absolute (*r* = −0.53, *P* < 0.001) and relative (*r* = −0.48, *P* < 0.001) liver weight correlated with liver mtDNA amount (Figure 4A). Indicators of muscle mass, including fetal weight (*r* = −0.29, *P* = 0.017), TC (*r* = −0.27, *P* = 0.027), CRL to TC ratio (CRL:TC) (*r* = 0.54, *P* < 0.001) and TC to rib length ratio (*r* = −0.47, *P* = 0.015), were also clearly correlated with mtDNA amount, while direct skeletal measurements analogous to TC and CRL:TC ratio, i.e., combined length of both sixth ribs and the ratio of spinal column length to combined length of both sixth ribs, showed no relationship (*P* > 0.05) (Figure 4B).

### Relationships Between Mitochondrial Gene Expression and Phenotype

**FIGURE 5:**
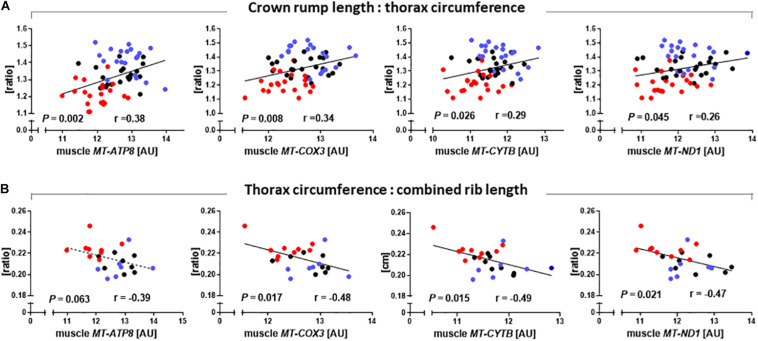
Relationships between phenotypic parameters that reveal increased muscle mass of somatic cell nuclear transfer (SCNT) fetuses and mitochondrial gene expression in skeletal muscle. **(A)** Ratio of crown rump length to thorax circumference and **(B)** ratio of thorax circumference to combined rib length correlate with *MT-ATP8*, *MT-COX3*, *MT-CYTB*, and *MT-ND1* transcript abundances in muscle of SCNT (red), *in vitro* fertilization (IVF, blue), and artificial insemination (AI, black) fetuses. Regressions are shown with Pearson correlation coefficients (r) and *P-*values. Mitochondrial gene expression is expressed in arbitrary units (AU).

As SCNT affected mitochondrial gene expression in skeletal muscle, we also tested relationships between major phenotypic indicators of increased muscle mass and mtDNA transcript abundances. CRL:TC was significantly correlated with *MT-ATP8* (*r* = 0.38, *P* = 0.002), *MT-COX3* (*r* = 0.34, *P* = 0.008), *MT-CYTB* (*r* = 0.29, *P* = 0.026), and *MT-ND1* (*r* = 0.26, *P* = 0.045) transcript (Figure 5A). TC to combined rib length ratio was similarly correlated with *MT-COX3* (*r* = 0.48, *P* = 0.017), *MT-CYTB* (*r* = 0.49, *P* = 0.015), and *MT-ND1* (*r* = 0.47, *P* = 0.021) transcript abundance, while *MT-ATP8* (*r* = 0.39, *P* = 0.063) approached significance (Figure 5B).

## Discussion

We discovered mtDNA depletion in skeletal muscle of viable bovine day 80 SCNT fetuses compared with AI and IVF controls. Results obtained with a highly standardized core subset of fetuses were consistent with results obtained for the larger, more heterogeneous entire dataset, which increased statistical power and provided additional evidence for mtDNA depletion in SCNT liver. The reduction in mtDNA amount was clearly attributable to SCNT procedures and not the *in vitro* embryo environment *per se*, as mtDNA amount in fetuses derived from IVF embryos cultured under the same conditions as SCNT embryos was unaffected. The unique effects of SCNT were also evident from the abnormal SCNT phenotype with hepatomegaly and muscle hypertrophy that is distinct from the proportionate increase in size of otherwise normal IVF fetuses.

The lack of changes in mtDNA amount of the SCNT brain and the magnitude of mtDNA depletion in SCNT muscle (−55%) and liver (−56%), which is much higher than expected from the ∼10% of ooplasm that is typically removed in enucleation (Hua et al., 2011), may be explained by specific aspects of mitochondrial distribution and mtDNA replication in the oocyte and embryo. In the early mouse MII oocyte, mitochondrial distribution is nonrandom with significant accumulation of the organelles around the meiotic spindle (Nagai et al., 2006; Dalton and Carroll, 2013). Removal of the MII spindle by enucleation thus eliminates a disproportionately large quantity of mitochondria from the oocyte. Furthermore, as demonstrated in the reconstructed SCNT pig embryo, removal of the spindle disrupts cytoskeletal dynamics and translocation of remaining mitochondria to the perinuclear region (Katayama et al., 2006). The perinuclear distribution of mitochondria is crucial for partitioning of the organelles to blastomeres and can affect the proportion of mitochondria allocated to daughter cells (Van Blerkom et al., 2000). Distribution of mitochondria to daughter cells in early cell divisions is unequal (Van Blerkom et al., 2000), and mtDNA content of individual blastomeres differs by an order of magnitude between the two- and eight-cell stage in human and pig (Lin et al., 2004; El Shourbagy et al., 2006). It has been speculated that unequal partitioning of mitochondria and mtDNA to blastomeres might affect differentiation and development due to essential mitochondrial functions in energy production, cell signaling, and apoptosis (Kameyama et al., 2010). More recent data obtained in mouse have indeed implicated mitochondrial 16S rRNA in cell lineage allocation of blastomeres as early as the two-cell stage (Zheng et al., 2016). This is of particular interest in the context of species-specific windows of mtDNA turnover and replication in the preimplantation embryo that may be adversely impacted by SCNT. Overall, mtDNA copy number in the preimplantation mouse embryo has been reported as stable (Piko and Taylor, 1987; Ebert et al., 1988; Thundathil et al., 2005; Aiken et al., 2008), but there is a period of rapid mtDNA turnover and replication immediately after fertilization (McConnell and Petrie, 2004). At this developmental stage, mtDNA amount is susceptible to environmental perturbation, and any reduction in mtDNA may persist into the fetal stage and beyond (McConnell and Petrie, 2004). Similar to mouse, mtDNA turnover and replication in pig and human involve mtDNA reduction between the oocyte and the two-cell stage followed by a significant increase in mtDNA at the blastocyst stage (Spikings et al., 2007; Hashimoto et al., 2017). In bovine, a 60% reduction in mtDNA amount after the two-cell stage is followed by a marked increase in mtDNA at the blastocyst stage (May-Panloup et al., 2005). While mitochondrial transcripts have so far only been implicated in lineage formation of trophectoderm (Zheng et al., 2016), it has been demonstrated that two cells of a four-cell embryo typically contribute to the resulting mouse, and in fact, individual 16-cell and occasionally even 32-cell mouse blastomeres can sporadically generate a complete animal (Tarkowski et al., 2001, 2010). Furthermore, at most eight cells of the epiblast contribute to the somatic lineages of the mouse embryo (Soriano and Jaenisch, 1986). It is noteworthy that the three fetal tissues investigated here are derived from different germ layers and show either no (brain) or a significant (liver, muscle) reduction in mtDNA amount. A disproportionate reduction in mtDNA through removal of the perinuclear ooplasm, perturbation of mitochondrial distribution to embryonic cells, and adverse effects of SCNT on mtDNA turnover and replication in the reconstructed embryo at potentially crucial time points for lineage formation may thus explain the pronounced but tissue-specific mtDNA depletion observed in the first-trimester bovine SCNT fetus. This is especially true for tissues that have a high proliferative capacity and hence mtDNA requirement to fuel their stem cell niche (Chen et al., 2020).

Embryo–fetal organ development depends on stringent thresholds for mtDNA amount and transcript levels that are determined by tissue- and developmental stage-specific energy demand (Heerdt and Augenlicht, 1990; Larsson et al., 1998; Huo and Scarpulla, 2001). Developing mitotic (liver) and postmitotic (muscle) tissues differ in energy demand, mtDNA amount, and control of mitochondrial biogenesis (Pejznochova et al., 2010). The divergent pattern in mtDNA transcript abundances, i.e., significant reduction in muscle only, suggests an organ-specific requirement and/or ability to restore adequate amounts of mtDNA transcript through compensatory mechanisms. The liver develops earlier than the skeletal muscle, has initially important functions in hematopoiesis (Zaret, 2002; Buckingham et al., 2003), and has a much higher turnover of respiratory chain enzyme proteins than the muscle (Kim et al., 2012); restoration of liver mtDNA transcript may thus be a requirement for embryo–fetal survival.

 In our bovine resource, indicators of fetal muscle mass were inversely correlated with mtDNA amount and mtDNA transcript abundance. MtDNA depletion and associated mitochondria to nucleus stress signaling has been shown to significantly increase expression of *TGF*β and induce tumorigenic capacity of myoblasts *in vitro* (Amuthan et al., 2001). Our data for skeletal muscle are further supported by mouse models where mtDNA depletion caused by *MSTN* (Amthor et al., 2007) or *THRA* (Pessemesse et al., 2012) ablation is associated with increased muscle mass. Moreover, fiber composition and structure in mtDNA-depleted mouse muscle was altered, and muscle strength was diminished (Amthor et al., 2007). This is consistent with altered fiber composition and structure in fetal and postnatal bovine SCNT muscle (Jurie et al., 2009; Cassar-Malek et al., 2010) and muscle hypotonia in SCNT offspring (e.g., Hill, 2014) and humans with mtDNA depletion syndrome (e.g., Nogueira et al., 2014).

DNA hypermethylation and lower transcript abundance of the gene encoding mtDNA polymerase γ catalytic subunit A (*POLGA*) was previously reported to be associated with mtDNA depletion in mouse SCNT-ESCs and interpreted as evidence for nuclear epigenetic reprogramming effects on mtDNA amount (Kelly et al., 2012). We found neither differences in expression levels of *POLGA* nor *POLGB* in bovine SCNT liver or muscle tissue with mtDNA depletion. We further tested expression levels of mitochondrial TFAM, which is crucial for mtDNA replication and transcription but again found no differences in transcript levels of SCNT tissues and AI or IVF controls. These data argue against a nuclear epigenetic reprogramming effect on mtDNA and mtDNA transcript abundance. However, we cannot exclude that additional nuclear genes involved in the control of mtDNA copy number are affected by epigenetic reprogramming defects.

We propose that SCNT-induced mtDNA depletion is a core principle of perturbations after SCNT that underlies, or contributes to, abnormal epigenetic features and phenotypes observed after SCNT (Kang et al., 2001; Santos et al., 2003; Beaujean et al., 2004; Hiendleder et al., 2004a; Liu et al., 2008; Chan et al., 2012; Zhang et al., 2014, 2016; Niemann, 2016). This concept is supported by a growing body of evidence that links mitochondria with epigenetic modification in the nucleus (Wallace and Fan, 2010; Minocherhomji et al., 2012; Castegna et al., 2015; Weinhouse, 2017). In cell culture models, mtDNA depletion is associated with reversible changes in DNA methylation patterns of nuclear genes (Smiraglia et al., 2008). Moreover, progressive mitochondrial dysfunction induced by mtDNA depletion leads to metabolic and transcriptional changes that affect methionine metabolism and DNA methylation (Lozoya et al., 2018). Changes in mitochondrial redox state and metabolites have also been associated with changes in histone modification and transcriptome profile (Kopinski et al., 2019). Finally, and further supporting our hypothesis, bovine SCNT blastocysts were recently shown to have a significantly lower mitochondrial mass than expected from the amount of ooplasm removed by enucleation and display increased abundances of DNA methyl transferase (*DNMT1* and *DNMT3A*) transcripts as compared with IVF controls (Xu et al., 2019).

In conclusion, our findings provide the basis for exploring compromised epigenetic reprogramming of the SCNT embryo in the context of earlier, mitochondrial perturbation during the embryo reconstruction process that may affect nuclear reprogramming. This provides a novel perspective on the reprogramming process and opens new avenues to improve SCNT protocols for healthy embryo and tissue development.

## Data Availability Statement

The original contributions presented in the study are included in the article/Supplementary Material, further inquiries can be directed to the corresponding author/s.

## Ethics Statement

The animal study was reviewed and approved by the Regierung von Oberbayern (Bavaria, Germany).

## Author Contributions

DB: investigation, formal analysis, validation, visualization, funding acquisition, writing—original draft, and writing—review and editing. SU: methodology, investigation, funding acquisition, resources, and validation. KG: investigation and validation. VZ and MR: investigation, methodology, and validation. H-DR: investigation, methodology, supervision, and validation. PV: writing—original draft and writing—review and editing. EW: methodology, conceptualization, funding acquisition, resources, and supervision. SL: funding acquisition, writing—original draft, and writing—review and editing. SH: conceptualization, methodology, formal analysis, project administration, funding acquisition, resources, supervision, visualization, writing—original draft, and writing—review and editing. All authors have read and agreed to the published version of the manuscript.

## Conflict of Interest

MR is employed by the company Bayern-Genetik GmbH, Grub, Germany. The remaining authors declare that the research was conducted in the absence of any commercial or financial relationships that could be construed as a potential conflict of interest.

## References

[B1] Aiken C. E. Cindrova-Davies T. Johnson M. H. (2008). Variations in mouse mitochondrial DNA copy number from fertilization to birth are associated with oxidative stress. *Reprod. Biomed. Online* 17 806–813. 10.1016/S1472-6483(10)60409-9 19079965

[B2] Amthor H. Macharia R. Navarrete R. Schuelke M. Brown S. C. Otto A. (2007). Lack of myostatin results in excessive muscle growth but impaired force generation. *Proc. Natl. Acad. Sci. U.S.A.* 104 1835–1840. 10.1073/pnas.0604893104 17267614 PMC1794294

[B3] Amuthan G. Biswas G. Zhang S. Y. Klein-Szanto A. Vijayasarathy C. Avadhani N. G. (2001). Mitochondria-to-nucleus stress signaling induces phenotypic changes, tumor progression and cell invasion. *EMBO J.* 20 1910–1920. 10.1093/emboj/20.8.1910 11296224 PMC125420

[B4] Battersby B. J. Loredo-Osti J. C. Shoubridge E. A. (2003). Nuclear genetic control of mitochondrial DNA segregation. *Nat. Genet.* 33 183–186. 10.1038/ng1073 12539044

[B5] Beaujean N. Taylor J. Gardner J. Wilmut I. Meehan R. Young L. (2004). Effect of limited DNA methylation reprogramming in the normal sheep embryo on Somatic cell nuclear transfer. *Biol. Reprod.* 71 185–193. 10.1095/biolreprod.103.026559 14998909

[B6] Brisville A. C. Fecteau G. Boysen S. Desrochers A. Dorval P. Buczinski S. (2013). Neonatal morbidity and mortality of 31 calves derived from somatic cloning. *J. Vet. Intern. Med.* 27 1218–1227. 10.1111/jvim.12129 23782425

[B7] Bruggerhoff K. Zakhartchenko V. Wenigerkind H. Reichenbach H. D. Prelle K. Schernthaner W. (2002). Bovine somatic cell nuclear transfer using recipient oocytes recovered by ovum pick-up: effect of maternal lineage of oocyte donors. *Biol. Reprod.* 66 367–373. 10.1095/biolreprod66.2.367 11804950

[B8] Buckingham M. Bajard L. Chang T. Daubas P. Hadchouel J. Meilhac S. (2003). The formation of skeletal muscle: from somite to limb. *J. Anat.* 202 59–68. 10.1046/j.1469-7580.2003.00139.x 12587921 PMC1571050

[B9] Campbell K. H. McWhir J. Ritchie W. A. Wilmut I. (1996). Sheep cloned by nuclear transfer from a cultured cell line. *Nature* 380 64–66. 10.1038/380064a0 8598906

[B10] Cassar-Malek I. Picard B. Jurie C. Listrat A. Guillomot M. Chavatte-Palmer P. (2010). Myogenesis is delayed in bovine fetal clones. *Cell. Reprogram.* 12 191–201. 10.1089/cell.2009.0065 20677933

[B11] Castegna A. Iacobazzi V. Infantino V. (2015). The mitochondrial side of epigenetics. *Physiol. Genomics* 47 299–307. 10.1152/physiolgenomics.00096.2014 26038395

[B12] Chan M. M. Smith Z. D. Egli D. Regev A. Meissner A. (2012). Mouse ooplasm confers context-specific reprogramming capacity. *Nat. Genet.* 44 978–980. 10.1038/ng.2382 22902786 PMC3432711

[B13] Chavatte-Palmer P. Remy D. Cordonnier N. Richard C. Issenman H. Laigre P. (2004). Health status of cloned cattle at different ages. *Cloning Stem Cells* 6 94–100. 10.1089/1536230041372274 15268782

[B14] Chen J. Zheng Q. Peiffer L. B. Hicks J. L. Haffner M. C. Rosenberg A. Z. (2020). An in situ atlas of mitochondrial DNA in mammalian tissues reveals high content in stem and proliferative compartments. *Am. J. Pathol.* 190 1565–1579. 10.1016/j.ajpath.2020.03.018 32304697 PMC7338910

[B15] Chomczynski P. Sacchi N. (1987). Single-step method of RNA isolation by acid guanidinium thiocyanate-phenol-chloroform extraction. *Anal. Biochem.* 162 156–159. 10.1016/0003-2697(87)90021-2 2440339

[B16] Dalton C. M. Carroll J. (2013). Biased inheritance of mitochondria during asymmetric cell division in the mouse oocyte. *J. Cell Sci.* 126(Pt 13) 2955–2964. 10.1242/jcs.128744 23659999 PMC3699109

[B17] de Montera B. El Zeihery D. Müller S. Jammes H. Brem G. Reichenbach H. D. (2010). Quantification of leukocyte genomic 5-methylcytosine levels reveals epigenetic plasticity in healthy adult cloned cattle. *Cell. Reprogram.* 12 175–181. 10.1089/cell.2009.0062 20677931 PMC2993042

[B18] Ebert K. M. Liem H. Hecht N. B. (1988). Mitochondrial DNA in the mouse preimplantation embryo. *J. Reprod. Fertil.* 82 145–149. 10.1530/jrf.0.0820145 3339575

[B19] Edwards J. L. Schrick F. N. Hockett M. E. Saxton A. M. Lawrence J. L. Payton R. R. (2003). Development of clones constructed with maternal cytoplasm. *Theriogenology* 59:250.

[B20] El Shourbagy S. H. Spikings E. C. Freitas M. St John J. C. (2006). Mitochondria directly influence fertilisation outcome in the pig. *Reproduction* 131 233–245. 10.1530/rep.1.00551 16452717

[B21] El-Hattab A. W. Scaglia F. (2013). Mitochondrial DNA depletion syndromes: review and updates of genetic basis, manifestations, and therapeutic options. *Neurotherapeutics* 10 186–198. 10.1007/s13311-013-0177-6 23385875 PMC3625391

[B22] Finsterer J. Ahting U. (2013). Mitochondrial depletion syndromes in children and adults. *Can. J. Neurol. Sci.* 40 635–644. 10.1017/S0317167100014852 23968935

[B23] Hance N. Ekstrand M. I. Trifunovic A. (2005). Mitochondrial DNA polymerase gamma is essential for mammalian embryogenesis. *Hum. Mol. Genet.* 14 1775–1783. 10.1093/hmg/ddi184 15888483

[B24] Hashimoto S. Morimoto N. Yamanaka M. Matsumoto H. Yamochi T. Goto H. (2017). Quantitative and qualitative changes of mitochondria in human preimplantation embryos. *J. Assist. Reprod. Genet.* 34 573–580. 10.1007/s10815-017-0886-6 28190213 PMC5427646

[B25] Heerdt B. G. Augenlicht L. H. (1990). Changes in the number of mitochondrial genomes during human development. *Exp. Cell Res.* 186 54–59. 10.1016/0014-4827(90)90209-S 2153553

[B26] Hiendleder S. (2007). Mitochondrial DNA inheritance after SCNT. *Adv. Exp. Med. Biol.* 591 103–116. 10.1007/978-0-387-37754-4_8 17176558

[B27] Hiendleder S. Mund C. Reichenbach H. D. Wenigerkind H. Brem G. Zakhartchenko V. (2004a). Tissue-specific elevated genomic cytosine methylation levels are associated with an overgrowth phenotype of bovine fetuses derived by in vitro techniques. *Biol. Reprod.* 71 217–223. 10.1095/biolreprod.103.026062 15028629

[B28] Hiendleder S. Prelle K. Brüggerhoff K. Reichenbach H. D. Wenigerkind H. Bebbere D. (2004b). Nuclear-cytoplasmic interactions affect in utero developmental capacity, phenotype, and cellular metabolism of bovine nuclear transfer fetuses. *Biol. Reprod.* 70 1196–1205. 10.1095/biolreprod.103.023028 14681199

[B29] Hiendleder S. Zakhartchenko V. Wenigerkind H. Reichenbach H. D. Brüggerhoff K. Prelle K. (2003). Heteroplasmy in bovine fetuses produced by intra- and inter-subspecific somatic cell nuclear transfer: neutral segregation of nuclear donor mitochondrial DNA in various tissues and evidence for recipient cow mitochondria in fetal blood. *Biol. Reprod.* 68 159–166. 10.1095/biolreprod.102.008201 12493708

[B30] Hiendleder S. Zakhartchenko V. Wolf E. (2005). Mitochondria and the success of somatic cell nuclear transfer cloning: from nuclear-mitochondrial interactions to mitochondrial complementation and mitochondrial DNA recombination. *Reprod. Fertil. Dev.* 17 69–83. 10.1071/RD04115 15745633

[B31] Hill J. R. (2014). Incidence of abnormal offspring from cloning and other assisted reproductive technologies. *Annu. Rev. Anim. Biosci.* 2 307–321. 10.1146/annurev-animal-022513-114109 25384145

[B32] Hua S. Zhang H. Su J. M. Zhang T. Quan F. S. Liu J. (2011). Effects of the removal of cytoplasm on the development of early cloned bovine embryos. *Anim. Reprod. Sci.* 126 37–44. 10.1016/j.anireprosci.2011.05.002 21632190

[B33] Huo L. Scarpulla R. C. (2001). Mitochondrial DNA instability and peri-implantation lethality associated with targeted disruption of nuclear respiratory factor 1 in mice. *Mol. Cell Biol.* 21 644–654. 10.1128/MCB.21.2.644-654.2001 11134350 PMC86640

[B34] Jenuth J. P. Peterson A. C. Fu K. Shoubridge E. A. (1996). Random genetic drift in the female germline explains the rapid segregation of mammalian mitochondrial DNA. *Nat. Genet.* 14 146–151. 10.1038/ng1096-146 8841183

[B35] Jiao F. Yan J. B. Yang X. Y. Li H. Wang Q. Huang S. Z. (2007). Effect of oocyte mitochondrial DNA haplotype on bovine somatic cell nuclear transfer efficiency. *Mol. Reprod. Dev.* 74 1278–1286. 10.1002/mrd.20698 17290429

[B36] Jurie C. Picard B. Heyman Y. Cassar-Malek I. Chavatte-Palmer P. Richard C. (2009). Comparison of cloned and non-cloned Holstein heifers in muscle contractile and metabolic characteristics. *Animal* 3 244–250. 10.1017/S1751731108003406 22444227

[B37] Kameyama Y. Ohnishi H. Shimoi G. Hashizume R. Ito M. Smith L. C. (2010). Asymmetrical allocation of mitochondrial DNA to blastomeres during the first two cleavages in mouse embryos. *Reprod. Fertil. Dev.* 22 1247–1253. 10.1071/RD10076 20883650

[B38] Kang Y. K. Koo D. B. Park J. S. Choi Y. H. Chung A. S. Lee K. K. (2001). Aberrant methylation of donor genome in cloned bovine embryos. *Nat. Genet.* 28 173–177. 10.1038/88903 11381267

[B39] Katayama M. Zhong Z. Lai L. Sutovsky P. Prather R. S. Schatten H. (2006). Mitochondrial distribution and microtubule organization in fertilized and cloned porcine embryos: implications for developmental potential. *Dev. Biol.* 299 206–220. 10.1016/j.ydbio.2006.07.022 16945363 PMC1852431

[B40] Kelly R. D. Mahmud A. McKenzie M. Trounce I. A. St John J. C. (2012). Mitochondrial DNA copy number is regulated in a tissue specific manner by DNA methylation of the nuclear-encoded DNA polymerase gamma A. *Nucleic Acids Res.* 40 10124–10138. 10.1093/nar/gks770 22941637 PMC3488228

[B41] Kim T. Y. Wang D. Kim A. K. Lau E. Lin A. J. Liem D. A. (2012). Metabolic labeling reveals proteome dynamics of mouse mitochondria. *Mol. Cell. Proteomics* 11 1586–1594. 10.1074/mcp.M112.021162 22915825 PMC3518123

[B42] Kopinski P. K. Janssen K. A. Schaefer P. M. Trefely S. Perry C. E. Potluri P. (2019). Regulation of nuclear epigenome by mitochondrial DNA heteroplasmy. *Proc. Natl. Acad. Sci. U.S.A.* 116 16028–16035. 10.1073/pnas.1906896116 31253706 PMC6689928

[B43] Larsson N. G. Wang J. Wilhelmsson H. Oldfors A. Rustin P. Lewandoski M. (1998). Mitochondrial transcription factor a is necessary for mtDNA maintenance and embryogenesis in mice. *Nat. Genet.* 18 231–236. 10.1038/ng0398-231 9500544

[B44] Lee E. Song K. (2007). Autologous somatic cell nuclear transfer in pigs using recipient oocytes and donor cells from the same animal. *J. Vet. Sci.* 8 415–421. 10.4142/jvs.2007.8.4.415 17993757 PMC2868159

[B45] Lin D. P. Huang C. C. Wu H. M. Cheng T. C. Chen C. I. Lee M. S. (2004). Comparison of mitochondrial DNA contents in human embryos with good or poor morphology at the 8-cell stage. *Fertil. Steril.* 81 73–79. 10.1016/j.fertnstert.2003.05.005 14711547

[B46] Liu J. H. Yin S. Xiong B. Hou Y. Chen D. Y. Sun Q. Y. (2008). Aberrant DNA methylation imprints in aborted bovine clones. *Mol. Reprod. Dev.* 75 598–607. 10.1002/mrd.20803 17886268

[B47] Liu Z. Cai Y. Wang Y. Nie Y. Zhang C. Xu Y. (2018). Cloning of macaque monkeys by Somatic cell nuclear transfer. cell. 2018;172(4):881-887 e7. *Erratum Cell* 174:245. 10.1016/j.cell.2018.01.020 29395327

[B48] Lozoya O. A. Martinez-Reyes I. Wang T. Grenet D. Bushel P. Li J. (2018). Mitochondrial nicotinamide adenine dinucleotide reduced (n.d.) oxidation links the tricarboxylic acid (TCA) cycle with methionine metabolism and nuclear DNA methylation. *PLoS Biol.* 16:e2005707. 10.1371/journal.pbio.2005707 29668680 PMC5927466

[B49] Matoba S. Zhang Y. (2018). Somatic cell nuclear transfer reprogramming: mechanisms and applications. *Cell Stem Cell* 23 471–485. 10.1016/j.stem.2018.06.018 30033121 PMC6173619

[B50] May-Panloup P. Vignon X. Chrétien M. F. Heyman Y. Tamassia M. Malthièry Y. (2005). Increase of mitochondrial DNA content and transcripts in early bovine embryogenesis associated with upregulation of mtTFA and NRF1 transcription factors. *Reprod. Biol. Endocrinol.* 3:65. 10.1186/1477-7827-3-65 16285882 PMC1298334

[B51] McConnell J. M. L. Petrie L. (2004). Mitochondrial DNA turnover occurs during preimplantation development and can be modulated by environmental factors. *Reprod. Biomed. Online* 9 418–424. 10.1016/S1472-6483(10)61277-1 15511342

[B52] Minocherhomji S. Tollefsbol T. O. Singh K. K. (2012). Mitochondrial regulation of epigenetics and its role in human diseases. *Epigenetics* 7 326–334. 10.4161/epi.19547 22419065 PMC3368816

[B53] Murakami M. Perez O. Ferguson C. E. Behboodi E. Denniston R. S. Godke R. A. (2003). Use of in vivo-recovered oocytes and adult somatic cells from the same donor for nuclear transfer in cattle. *Vet. Rec.* 153 713–714. 14690076

[B54] Nagai S. Mabuchi T. Hirata S. Shoda T. Kasai T. Yokota S. (2006). Correlation of abnormal mitochondrial distribution in mouse oocytes with reduced developmental competence. *Tohoku J. Exp. Med.* 210 137–144. 10.1620/tjem.210.137 17023767

[B55] Niemann H. (2016). Epigenetic reprogramming in mammalian species after SCNT-based cloning. *Theriogenology* 86 80–90. 10.1016/j.theriogenology.2016.04.021 27160443

[B56] Niemann H. Lucas-Hahn A. (2012). Somatic cell nuclear transfer cloning: practical applications and current legislation. *Reprod. Domest. Anim.* 47(Suppl. 5) 2–10. 10.1111/j.1439-0531.2012.02121.x 22913555

[B57] Nogueira C. Almeida L. S. Nesti C. Pezzini I. Videira A. Vilarinho L. (2014). Syndromes associated with mitochondrial DNA depletion. *Ital. J. Pediatr.* 40:34. 10.1186/1824-7288-40-34 24708634 PMC3985578

[B58] Pejznochova M. Tesarova M. Hansikova H. Magner M. Honzik T. Vinsova K. (2010). Mitochondrial DNA content and expression of genes involved in mtDNA transcription, regulation and maintenance during human fetal development. *Mitochondrion* 10 321–329. 10.1016/j.mito.2010.01.006 20096380

[B59] Pessemesse L. Schlernitzauer A. Sar C. Levin J. Grandemange S. Seyer P. (2012). Depletion of the p43 mitochondrial T3 receptor in mice affects skeletal muscle development and activity. *FASEB J.* 26 748–756. 10.1096/fj.11-195933 22109994

[B60] Piko L. Taylor K. D. (1987). Amounts of mitochondrial DNA and abundance of some mitochondrial gene transcripts in early mouse embryos. *Dev. Biol.* 123 364–374. 10.1016/0012-1606(87)90395-2 2443405

[B61] Rhind S. M. Taylor J. E. De Sousa P. A. King T. J. McGarry M. Wilmut I. (2003). Human cloning: can it be made safe? *Nat. Rev. Genet.* 4 855–864. 10.1038/nrg1205 14634633

[B62] Roubertoux P. L. Sluyter F. Carlier M. Marcet B. Maarouf-Veray F. Chérif C. (2003). Mitochondrial DNA modifies cognition in interaction with the nuclear genome and age in mice. *Nat. Genet.* 35 65–69. 10.1038/ng1230 12923532

[B63] Sakai R. R. Tamashiro K. L. Yamazaki Y. Yanagimachi R. (2005). Cloning and assisted reproductive techniques: influence on early development and adult phenotype. *Birth Defects Res. C Embryo Today* 75 151–162. 10.1002/bdrc.20042 16035042

[B64] Santos F. Zakhartchenko V. Stojkovic M. Peters A. Jenuwein T. Wolf E. (2003). Epigenetic marking correlates with developmental potential in cloned bovine preimplantation embryos. *Curr. Biol.* 13 1116–1121. 10.1016/S0960-9822(03)00419-6 12842010

[B65] Sarzi E. Bourdon A. Chrétien D. Zarhrate M. Corcos J. Slama A. (2007). Mitochondrial DNA depletion is a prevalent cause of multiple respiratory chain deficiency in childhood. *J Pediatr.* 150 531–534, 534.e1-6. 10.1016/j.jpeds.2007.01.044 17452231

[B66] Sharpley M. S. Marciniak C. Eckel-Mahan K. McManus M. Crimi M. Waymire K. (2012). Heteroplasmy of mouse mtDNA is genetically unstable and results in altered behavior and cognition. *Cell* 151 333–343. 10.1016/j.cell.2012.09.004 23063123 PMC4175720

[B67] Shen C. J. Lin C. C. Shen P. C. Cheng W. T. Chen H. L. Chang T. C. (2013). Imprinted genes and satellite loci are differentially methylated in bovine somatic cell nuclear transfer clones. *Cell. Reprogram.* 15 413–424. 10.1089/cell.2013.0012 23961768 PMC3787327

[B68] Smiraglia D. J. Kulawiec M. Bistulfi G. L. Gupta S. G. Singh K. K. (2008). A novel role for mitochondria in regulating epigenetic modification in the nucleus. *Cancer Biol. Ther.* 7 1182–1190. 10.4161/cbt.7.8.6215 18458531 PMC2639623

[B69] Soriano P. Jaenisch R. (1986). Retroviruses as probes for mammalian development: allocation of cells to the somatic and germ cell lineages. *Cell* 46 19–29. 10.1016/0092-8674(86)90856-1 3013418

[B70] Spikings E. C. Alderson J. St John J. C. (2007). Regulated mitochondrial DNA replication during oocyte maturation is essential for successful porcine embryonic development. *Biol. Reprod.* 76 327–335. 10.1095/biolreprod.106.054536 17035641

[B71] Suomalainen A. Isohanni P. (2010). Mitochondrial DNA depletion syndromes–many genes, common mechanisms. *Neuromuscul. Disord.* 20 429–437. 10.1016/j.nmd.2010.03.017 20444604

[B72] Tachibana M. Amato P. Sparman M. Gutierrez N. M. Tippner-Hedges R. Ma H. (2013). Human embryonic stem cells derived by somatic cell nuclear transfer. *Cell* 153 1228–1238. 10.1016/j.cell.2013.05.006 23683578 PMC3772789

[B73] Tan W. Proudfoot C. Lillico S. G. Whitelaw C. B. (2016). Gene targeting, genome editing: from Dolly to editors. *Transgenic Res.* 25 273–287. 10.1007/s11248-016-9932-x 26847670 PMC4882362

[B74] Tarkowski A. K. Ozdzeński W. Czołowska R. (2001). How many blastomeres of the 4-cell embryo contribute cells to the mouse body? *Int. J. Dev. Biol.* 45 811–816. 11732840

[B75] Tarkowski A. K. Suwińska A. Czołowska R. Ożdżeński W. (2010). Individual blastomeres of 16- and 32-cell mouse embryos are able to develop into foetuses and mice. *Dev. Biol.* 348 190–198. 10.1016/j.ydbio.2010.09.022 20932967

[B76] Thundathil J. Filion F. Smith L. C. (2005). Molecular control of mitochondrial function in preimplantation mouse embryos. *Mol. Reprod. Dev.* 71 405–413. 10.1002/mrd.20260 15895466

[B77] Van Blerkom J. Davis P. Alexander S. (2000). Differential mitochondrial distribution in human pronuclear embryos leads to disproportionate inheritance between blastomeres: relationship to microtubular organization. ATP content and competence. *Hum. Reprod.* 15 2621–2633. 10.1093/humrep/15.12.2621 11098036

[B78] von Kleist-Retzow J. C. Cormier-Daire V. Viot G. Goldenberg A. Mardach B. Amiel J. (2003). Antenatal manifestations of mitochondrial respiratory chain deficiency. *J. Pediatr.* 143 208–212. 10.1067/S0022-3476(03)00130-6 12970634

[B79] Wallace D. C. Fan W. (2010). Energetics, epigenetics, mitochondrial genetics. *Mitochondrion* 10 12–31. 10.1016/j.mito.2009.09.006 19796712 PMC3245717

[B80] Weinhouse C. (2017). Mitochondrial-epigenetic crosstalk in environmental toxicology. *Toxicology* 391 5–17. 10.1016/j.tox.2017.08.008 28855114 PMC5681427

[B81] Wells D. N. Forsyth J. T. McMillan V. Oback B. (2004). The health of somatic cell cloned cattle and their offspring. *Cloning Stem Cells* 6 101–110. 10.1089/1536230041372300 15268783

[B82] Wilmut I. Schnieke A. E. McWhir J. Kind A. J. Campbell K. H. (1997). Viable offspring derived from fetal and adult mammalian cells. *Nature* 385 810–813. 10.1038/385810a0 9039911

[B83] Xiang R. Lee A. M. Eindorf T. Javadmanesh A. Ghanipoor-Samami M. Gugger M. (2014). Widespread differential maternal and paternal genome effects on fetal bone phenotype at mid-gestation. *J. Bone Miner. Res.* 29 2392–2404. 10.1002/jbmr.2263 24753181

[B84] Xu L. Mesalam A. Lee K. L. Song S. H. Khan I. Chowdhury M. M. R. (2019). Improves the in vitro developmental competence and reprogramming efficiency of cloned bovine embryos by additional complimentary cytoplasm. *Cell. Reprogram.* 21 51–60. 10.1089/cell.2018.0050 30735075 PMC6383574

[B85] Xue F. Tian X. C. Du F. Kubota C. Taneja M. Dinnyes A. (2002). Aberrant patterns of X chromosome inactivation in bovine clones. *Nat. Genet.* 31 216–220. 10.1038/ng900 12032569

[B86] Yan Z. H. Zhou Y. Y. Fu J. Jiao F. Zhao L. W. Guan P. F. (2010). Donor-host mitochondrial compatibility improves efficiency of bovine somatic cell nuclear transfer. *BMC Dev. Biol.* 10:31. 10.1186/1471-213X-10-31 20302653 PMC2858029

[B87] Yang X. Y. Li H. Ma Q. W. Yan J. B. Zhao J. G. Li H. W. (2006). Improved efficiency of bovine cloning by autologous somatic cell nuclear transfer. *Reproduction* 132 733–739. 10.1530/rep.1.01118 17071774

[B88] Zaret K. S. (2002). Regulatory phases of early liver development: paradigms of organogenesis. *Nat. Rev. Genet.* 3 499–512. 10.1038/nrg837 12094228

[B89] Zhang S. Chen X. Wang F. An X. Tang B. Zhang X. (2016). Aberrant DNA methylation reprogramming in bovine SCNT preimplantation embryos. *Sci. Rep.* 6:30345. 10.1038/srep30345 27456302 PMC4960566

[B90] Zhang X. Wang D. Han Y. Duan F. Lv Q. Li Z. (2014). Altered imprinted gene expression and methylation patterns in mid-gestation aborted cloned porcine fetuses and placentas. *J. Assist. Reprod. Genet.* 31 1511–1517. 10.1007/s10815-014-0320-2 25172095 PMC4389936

[B91] Zheng Z. Li H. Zhang Q. Yang L. Qi H. (2016). Unequal distribution of 16S mtrRNA at the 2-cell stage regulates cell lineage allocations in mouse embryos. *Reproduction* 151 351–367. 10.1530/REP-15-0301 26762401

