# Supplementary material for: Mitochondrial DNA Depletion in Granulosa Cell Derived Nuclear Transfer Tissues
Source: Front Cell Dev Biol. 2021 May 14;9:664099. doi: 10.3389/fcell.2021.664099 (PMC8194821; doi:10.3389/fcell.2021.664099)
Supplement: Supplementary file 1 [file Data_Sheet_1.docx]

**Supplementary Table 1**. Detailed description of the entire sample set of tissues from Day 80 fetuses produced by artificial insemination (AI; n = 24), *in vitro* fertilization (IVF; n = 21) or somatic cell nuclear transfer (SCNT; n = 22). Sex: F - female, M - male. Genetics: BS – Brown Swiss, SI – Simmental. Available tissue samples are indicated by check mark ✓. Samples from Brown Swiss singleton fetuses included in the more stringently defined core subsets (see methods) to perform highly standardized comparisons between all female SCNT (n = 14) and AI (n = 9), and between male and female IVF (n = 9) and AI (n = 13) tissues, are indicated by the check mark ✓.

| No. | Treatment | Brain | Liver | | Muscle | | Genetics | Sex | Singleton | Included in core set |
| --- | --- | --- | --- | --- | --- | --- | --- | --- | --- | --- |
|  |  | **DNA** | **DNA** | **RNA** | **DNA** | **RNA** |  |  |  |  |
| 1 | AI | ✓ | ✓ | ✓ | ✓ | ✓ | BS | F | ✓ | ✓ |
| 2 | AI | ✓ | ✓ | ✓ | ✓ | ✓ | BS | F | ✓ | ✓ |
| 3 | AI | ✓ | ✓ | ✓ | ✓ | ✓ | BS | F | ✓ | ✓ |
| 4 | AI | ✓ | ✓ | ✓ | ✓ | ✓ | BS | M | ✓ | ✓ |
| 5 | AI | ✓ | ✓ | ✓ | ✓ | ✓ | BS | F | ✓ | ✓ |
| 6 | AI | ✓ | ✓ | ✓ | ✓ | ✓ | BS | M | ✓ | ✓ |
| 7 | AI | ✓ | ✓ | ✓ | ✓ | ✓ | BS | F | ✓ | ✓ |
| 8 | AI | ✓ | ✓ | ✓ | ✓ | ✓ | BS | F | ✓ | ✓ |
| 9 | AI | ✓ | ✓ | ✓ | ✓ | ✓ | SI | F | ✓ |  |
| 10 | AI | ✓ | ✓ | ✓ | ✓ | ✓ | SI | F |  |  |
| 11 | AI | ✓ | ✓ | ✓ | ✓ | ✓ | SI | F |  |  |
| 12 | AI | ✓ | ✓ | ✓ | ✓ | ✓ | SI | M |  |  |
| 13 | AI | ✓ | ✓ | ✓ | ✓ |  | SI | F |  |  |
| 14 | AI | ✓ | ✓ | ✓ | ✓ | ✓ | SI | F |  |  |
| 15 | AI | ✓ | ✓ | ✓ | ✓ | ✓ | SI | M |  |  |
| 16 | AI | ✓ | ✓ | ✓ | ✓ | ✓ | SI | F |  |  |
| 17 | AI | ✓ | ✓ |  | ✓ |  | SI | F |  |  |
| 18 | AI | ✓ | ✓ | ✓ | ✓ | ✓ | SI | F |  |  |
| 19 | AI | ✓ | ✓ | ✓ | ✓ | ✓ | SI | M |  |  |
| 20 | AI | ✓ | ✓ | ✓ | ✓ |  | BS | F | ✓ | ✓ |
| 21 | AI | ✓ | ✓ | ✓ | ✓ | ✓ | BS | M | ✓ | ✓ |
| 22 | AI | ✓ | ✓ | ✓ | ✓ | ✓ | BS | F | ✓ | ✓ |
| 23 | AI | ✓ | ✓ | ✓ | ✓ | ✓ | BS | F | ✓ | ✓ |
| 24 | AI | ✓ | ✓ | ✓ | ✓ | ✓ | BS | M | ✓ | ✓ |
| 25 | SCNT | ✓ | ✓ | ✓ | ✓ | ✓ | BS | F | ✓ | ✓ |
| 26 | SCNT | ✓ | ✓ | ✓ | ✓ | ✓ | BS | F | ✓ | ✓ |
| 27 | SCNT | ✓ | ✓ | ✓ | ✓ | ✓ | BS | F |  |  |
| 28 | SCNT | ✓ | ✓ | ✓ | ✓ | ✓ | BS | F |  |  |
| 29 | SCNT | ✓ | ✓ | ✓ | ✓ | ✓ | BS | F | ✓ | ✓ |
| 30 | SCNT | ✓ | ✓ | ✓ | ✓ | ✓ | BS | F | ✓ | ✓ |

**Supplementary Table 1, continued**

| No. | Treatment | Brain | Liver | | Muscle | | Genetics | Sex | Singleton | Included in core set |
| --- | --- | --- | --- | --- | --- | --- | --- | --- | --- | --- |
|  |  | **DNA** | **DNA** | **RNA** | **DNA** | **RNA** |  |  |  |  |
| 31 | SCNT | ✓ | ✓ | ✓ | ✓ | ✓ | BS | F | ✓ | ✓ |
| 32 | SCNT | ✓ | ✓ | ✓ | ✓ |  | BS | F | ✓ | ✓ |
| 33 | SCNT | ✓ | ✓ | ✓ | ✓ | ✓ | BS | F | ✓ | ✓ |
| 34 | SCNT | ✓ | ✓ | ✓ | ✓ | ✓ | BS | F | ✓ | ✓ |
| 35 | SCNT | ✓ | ✓ | ✓ | ✓ | ✓ | BS | F | ✓ | ✓ |
| 36 | SCNT | ✓ | ✓ | ✓ | ✓ | ✓ | BS | F | ✓ | ✓ |
| 37 | SCNT | ✓ | ✓ | ✓ | ✓ | ✓ | BS | F | ✓ | ✓ |
| 38 | SCNT | ✓ | ✓ | ✓ | ✓ | ✓ | BS | F | ✓ | ✓ |
| 39 | SCNT | ✓ | ✓ |  | ✓ | ✓ | BS | F | ✓ | ✓ |
| 40 | SCNT |  |  | ✓ |  | ✓ | BS | F | ✓ |  |
| 41 | SCNT | ✓ | ✓ | ✓ | ✓ | ✓ | BS | F | ✓ | ✓ |
| 42 | SCNT | ✓ | ✓ | ✓ | ✓ | ✓ | BS | F |  |  |
| 43 | SCNT | ✓ | ✓ | ✓ | ✓ | ✓ | BS | F |  |  |
| 44 | SCNT | ✓ | ✓ | ✓ | ✓ | ✓ | BS | F |  |  |
| 45 | SCNT | ✓ | ✓ | ✓ | ✓ | ✓ | BS | F |  |  |
| 46 | SCNT | ✓ | ✓ | ✓ | ✓ | ✓ | BS | F |  |  |
| 47 | IVF | ✓ | ✓ | ✓ | ✓ | ✓ | BS | F | ✓ | ✓ |
| 48 | IVF | ✓ | ✓ | ✓ | ✓ | ✓ | BS | F | ✓ | ✓ |
| 49 | IVF | ✓ | ✓ | ✓ | ✓ | ✓ | BS | F |  |  |
| 50 | IVF | ✓ | ✓ | ✓ | ✓ | ✓ | BS | F |  |  |
| 51 | IVF | ✓ | ✓ |  | ✓ | ✓ | BS | F | ✓ | ✓ |
| 52 | IVF | ✓ | ✓ | ✓ | ✓ | ✓ | BS | M | ✓ | ✓ |
| 53 | IVF | ✓ | ✓ |  | ✓ | ✓ | BS | M | ✓ | ✓ |
| 54 | IVF | ✓ | ✓ | ✓ | ✓ | ✓ | BS | M |  |  |
| 55 | IVF | ✓ | ✓ | ✓ | ✓ | ✓ | BS | F |  |  |
| 56 | IVF | ✓ | ✓ | ✓ | ✓ | ✓ | BS | M | ✓ | ✓ |
| 57 | IVF | ✓ | ✓ | ✓ | ✓ |  | BS | M |  |  |
| 58 | IVF | ✓ | ✓ | ✓ | ✓ | ✓ | BS | M |  |  |
| 59 | IVF | ✓ | ✓ | ✓ | ✓ | ✓ | BS | M | ✓ | ✓ |
| 60 | IVF | ✓ | ✓ | ✓ | ✓ | ✓ | BS | M |  |  |
| 61 | IVF | ✓ | ✓ | ✓ | ✓ | ✓ | BS | F |  |  |
| 62 | IVF | ✓ | ✓ | ✓ | ✓ | ✓ | BS | M |  |  |
| 63 | IVF | ✓ | ✓ | ✓ | ✓ | ✓ | BS | M |  |  |
| 64 | IVF | ✓ | ✓ | ✓ | ✓ | ✓ | BS | M | ✓ | ✓ |
| 65 | IVF | ✓ | ✓ | ✓ | ✓ | ✓ | BS | M |  |  |
| 66 | IVF | ✓ | ✓ | ✓ | ✓ | ✓ | BS | F |  |  |
| 67 | IVF | ✓ | ✓ | ✓ | ✓ | ✓ | BS | M | ✓ | ✓ |

**Supplementary Table 2.** Details of primers used for gene expression analysis by Real-Time PCR.

| **Gene** | **GenBank**  **accession n.** | **Primers** | **Annealing temperature** | **Amplicon (bp)** |
| --- | --- | --- | --- | --- |
| *ATP8* | NC_006853 | F: 5’ ACTAGACACGTCAACATGACTGA 3’  R: 5’ GTTGGTGTCAGTTCTGGATTG 3’ | 55° C | 117 |
| *COX3* | NC_006853 | F: 5’ GTCCCACTGCTCAACACCTCTG 3’  R: 5’ ATGCTTTCGGTCCCCTTCTATTA 3’ | 58° C | 90 |
| *CYTB* | NC_006853.1 | F: 5’ ACTACACATCCGACACAACAACAG 3’  R: 5’ GCTCCGTTTGCGTGTATGTATC 3’ | 58° C | 100 |
| *H3F3A* | NM_001014389.2 | F: 5’ ACTGGCTACAAAAGCCGCTC 3’  R: 5’ ACTTGCCTGGTGCAAAGCAC 3’ | 60° C | 233 |
| *ND1* | NC_006853.1 | F: 5′ AAGGTCCAAATGTCGTAGGTCC 3’  R: 5’ CTGAAGATGTAGCGGGTCGTAG 3’ | 58° C | 99 |
| *POLGA* | ENSBTAT00000011993 | F: 5' AACATGAAGGACATCCGTGAG 3'  R: 5' ACATGCCAGCCAGAGTCAC 3' | 60° C | 142 |
| *POLGB* | NM_001075191 | F: 5’ GTGCCTTGGAGCATTATGTT 3’  R: 5’ GAAGCTTCAGTCTTTTCACCG 3’ | 60° C | 151 |
| *TFAM* | NM_001034016 | F: 5’ CCAAGCTATGGAGGGAACTTC 3’  R: 5’ TCCAAAGATACCATTTGACTTGG 3’ | 60° C | 136 |
| *UBB* | NM_174133.2 | F: 5’ AGATCCAGGATAAGGAAGGCAT 3’  R: 5’ GCTCCACCTCCAGGGTGAT 3’ | 62° C | 198 |
| *YWHAZ* | NM_174814 | F: 5' AGGCTGAGCGATATGATGAC 3'  R: 5' GACCCTCCAAGATGACCTAC 3' | 60° C | 140 |

A

B

SCNT

AI

**Supplementary figure 1.** Disproportionate fetal overgrowth after somatic cell nuclear transfer (SCNT) is distinct from proportionate overgrowth after in vitro fertilization (IVF) procedures and involves hepatomegaly and increased muscle mass.

Specific effects of (**A**) SCNT and (**B**) IVF on fetal phenotype as compared with artificial insemination (AI) controls at Day 80 of gestation. The analyses were performed on two core subsets of the entire data set to achieve highly standardized comparisons between female SCNT and AI, and between male and female IVF and AI, Brown Swiss singleton Day 80 fetuses. Crown-rump : Thorax is crown-rump length to thorax circumference ratio; Rib length is combined length of both 6^th^ ribs; Spine : Rib length is the ratio of the length of the spinal column to the combined length of both 6^th^ ribs; Thorax : Rib length is the ratio of thorax circumference to combined length of both 6^th^ ribs. Means ± standard error of the mean (SEM) and P-values for signiﬁcant differences are shown (*t*-test, *P*<0.05). The number of fetuses analyzed for each parameter is given within bars.

**
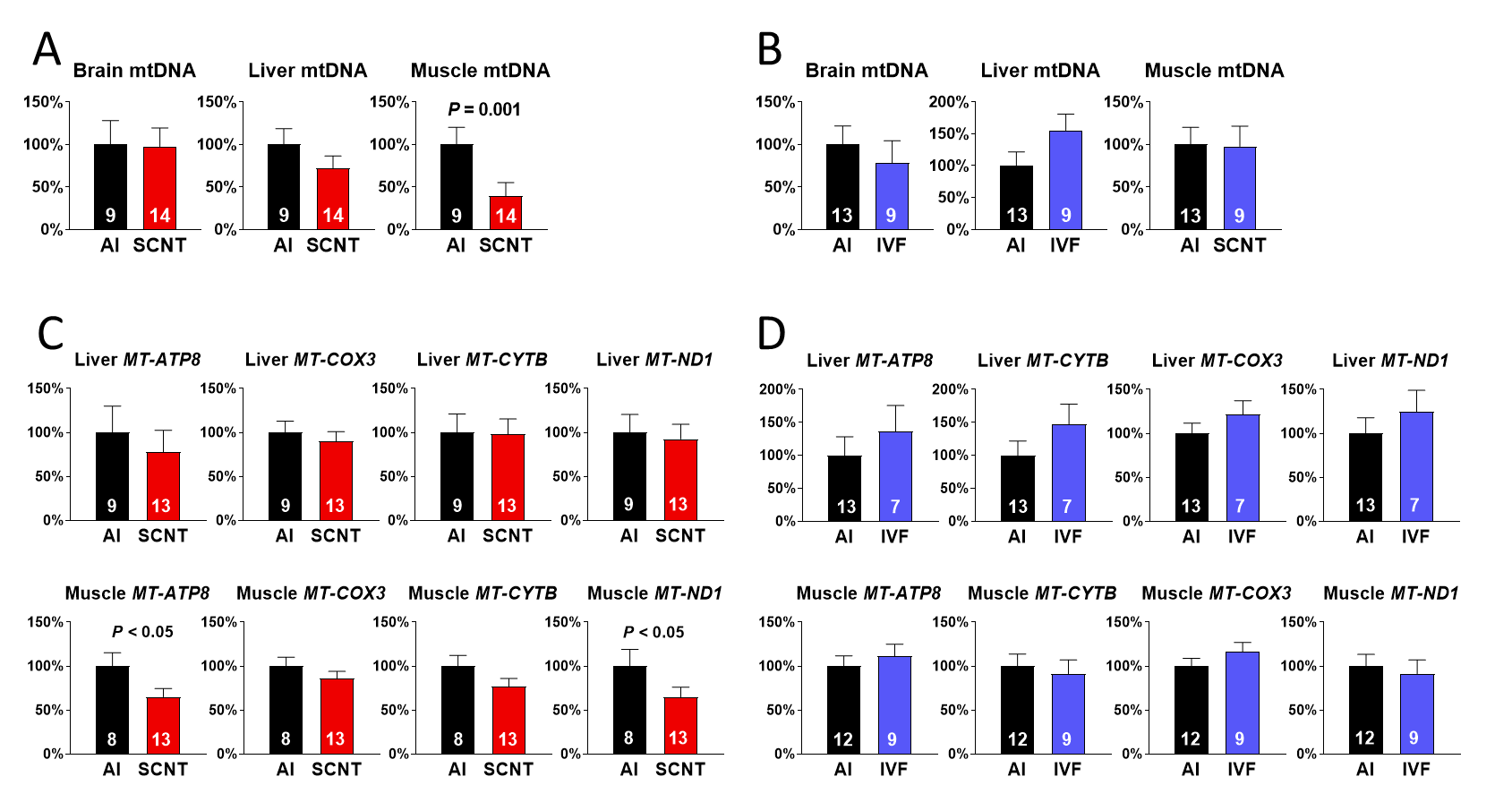
**

**Supplementary figure 2.** Somatic cell nuclear transfer (SCNT) but not *in vitro* fertilization (IVF) procedures affect mtDNA amount and mitochondrial gene expression.

Specific effects of (**A**) SCNT and (**B**) *in vitro* fertilization (IVF) on relative mtDNA amount of fetal tissues as compared with artificial insemination (AI) controls at Day 80 of gestation. Effects of SCNT (**C**) and IVF (**D**) as compared with AI on transcript abundance of mtDNA encoded subunits for respiratory chain enzyme complexes I, III, IV and V (*MT-ATP8, MT-COX3, MT-CYTB* and *MT-ND1*) in liver (top row) and skeletal muscle (bottom row). The analyses were performed on two core subsets of the entire data set to achieve highly standardized comparisons between female SCNT and AI, and between male and female IVF and AI, Brown Swiss singleton Day 80 fetuses. Means ± SEM and *P*-values for signiﬁcant differences are shown (*t*-test, *P*<0.05). The number of fetuses analyzed for each parameter is given within bars.

**
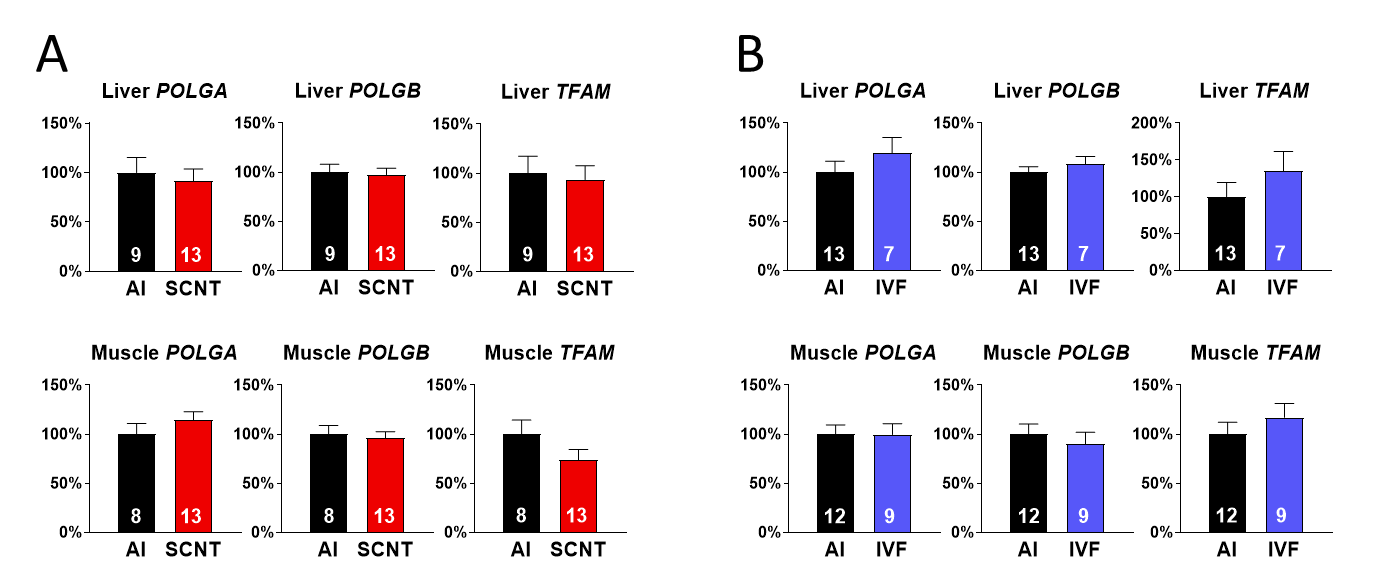
**

**Supplementary figure 3.** Somatic cell nuclear transfer (SCNT) does not affect the expression of nuclear-encoded genes pivotal for mtDNA replication and/or transcription.

Specific effects of (**A**) SCNT and (**B**) in vitro fertilization (IVF) on *POLGA, POLGB* and *TFAM* transcript abundance in fetal liver (top row) and skeletal muscle (bottom row) as compared with artificial insemination (AI) controls at Day 80 of gestation. The analyses were performed on two core subsets of the entire data set to achieve highly standardized comparisons between female SCNT and AI, and between male and female IVF and AI, Brown Swiss singleton Day 80 fetuses. Means ± SEM are shown, no signiﬁcant difference was found (*t*-test, *P*<0.05). The number of fetuses analyzed for each gene is given within bars.
